# A Case of Interstitial Cystitis With Hunner Lesions Involving Bilateral Ureters

**DOI:** 10.1002/iju5.70010

**Published:** 2025-04-29

**Authors:** Satoki Otsuka, Yoshiyuki Akiyama, Daichi Maeda, Aya Niimi, Kenichi Hashimoto, Jun Kamei, Satoru Taguchi, Yuta Yamada, Yusuke Sato, Daisuke Yamada, Tomonori Minagawa, Tetsuo Ushiku, Yukio Homma, Haruki Kume

**Affiliations:** ^1^ Department of Urology, Graduate School of Medicine The University of Tokyo Tokyo Japan; ^2^ Department of Urology Shinshu University School of Medicine Nagano Japan; ^3^ Department of Molecular and Cellular Pathology, Graduate School of Medical Sciences Kanazawa University Kanazawa Japan; ^4^ Department of Urology Tokyo Metropolitan Tama Medical Center Tokyo Japan; ^5^ Department of Pathology, Graduate School of Medicine The University of Tokyo Tokyo Japan; ^6^ Department of Interstitial Cystitis Medicine Kyorin University School of Medicine Tokyo Japan

**Keywords:** bladder pain syndrome, Hunner, hydronephrosis, IC/BPS, interstitial cystitis, stenosis, ureteral

## Abstract

**Introduction:**

Interstitial cystitis with Hunner lesion (IC/HL) is an enigmatic, chronic inflammatory disease of the urinary bladder. Few documented cases have reported the IC/HL involving the upper urinary tract.

**Case Presentation:**

A 51‐year‐old Japanese woman with IC/HL developed bilateral ureteral stenosis and associated hydronephrosis, resulting in increased serum creatinine concentrations. Cystography showed no evidence of bilateral vesicoureteral reflux or bladder deformity. Ureteroscopy showed severe ureteral stenosis due to mucosal hyperplasia at the mid/upper levels of both ureters, leading to the insertion of bilateral ureteral stents. Histological examination of the ureteral lesions showed similar chronic inflammatory changes to the bladder lesions, which were compatible with IC/HL. Bilateral ureteral stenosis persisted after a two‐year corticosteroid treatment (prednisolone, 7.5 mg/day), while IC/HL symptoms have disappeared.

**Conclusion:**

IC/HL could involve the upper ureters, and thus regular follow‐up imaging of upper urinary tracts in patients with IC/HL is warranted.

**Summary**

- The present report describes a rare case of IC/HL which developed bilateral ureteral IC/HL‐like inflammation and stenosis in addition to the bladder lesions despite the preservation of bladder capacity, suggesting that IC/HL inflammation could extend to the upper urinary tract.

## 1 Introduction

Interstitial cystitis with Hunner lesion (IC/HL) is an enigmatic, chronic inflammatory disease of the urinary bladder, characterized histologically by pancystitis, epithelial denudation, stromal fibrosis and edema, and dense subepithelial lymphoplasmacytic infiltration [1, 2, 3, 4]. IC/HL occurs usually in the urinary bladder, with few documented cases involving the upper urinary tract, although the extremely contracted end‐stage IC/HL bladder has been found to cause vesicoureteral reflux (VUR) with or without hydronephrosis [5]. The present report describes the rare case of a Japanese woman with IC/HL who simultaneously developed IC/HL‐like bilateral ureteral inflammation, which led to ureteral stenosis and associated hydronephrosis.

## 2 Clinical Summary

A 51‐year‐old woman with no previous history of pelvic organ diseases presented to a local urology clinic in 2019 with intractable bladder/urethral pain that worsened at bladder filling. Clinical examination excluded other bladder diseases that possibly cause similar symptoms such as acute cystitis and bladder calculi. Cystoscopy showed a Hunner lesion (HL) at the posterior bladder wall, which was electrocauterized, and the patient was subsequently treated with intravesical dimethyl sulfoxide at the local clinical providers, but her symptoms did not resolve. Computed tomography (CT) after a series of these treatments showed the development of bilateral upper/mid ureteral narrowing accompanied by hydronephrosis. Her serum creatinine levels increased gradually over the next 2 years, and she was referred in 2021 to our department for further causal investigations and treatment of her refractory IC/HL symptoms and bilateral ureteral strictures. Demographic data at her initial visit are shown in the Table 1.

**TABLE 1:** Baseline demographic and clinical characteristics of the patient analyzed in this study.

Hematology	
WBC	2.8 × 10^3^/μL
Hb	12.5 g/dL
Plt	45.0 × 10^6^/μL
Blood chemistry	
Cre	1.24 mg/dL
Estimated GFR	36.3 mL/min/1.73m^2^
CRP	0.42 mg/dL
Blood coagulation	
Fibrinogen	497 mg/dL
D‐dimer	1.7 μg/mL
Urine analysis	
WBC	> 100/HPF
RBC	50–99/HPF
Urine cultures and cytology	
Bacterial culture	No growth
Mycobacterial culture	No growth
Cytology	Class II
Others	
Anti‐ds‐DNA antibody	1.7 U/mL
Anti‐ss‐DNA antibody	14.1 U/mL
Anti‐SS‐A antibody	75.8 U/mL
Anti‐SS‐B antibody	0.9 U/mL
C3	136 mg/mL
C4	23 mg/mL
sIL‐2R	631 U/mL
TSH	2.49 μIU/mL
FT4	0.92 ng/dL
IgG	1737 mg/dL
IgG4	50.8 mg/dL

**FIGURE 1:**
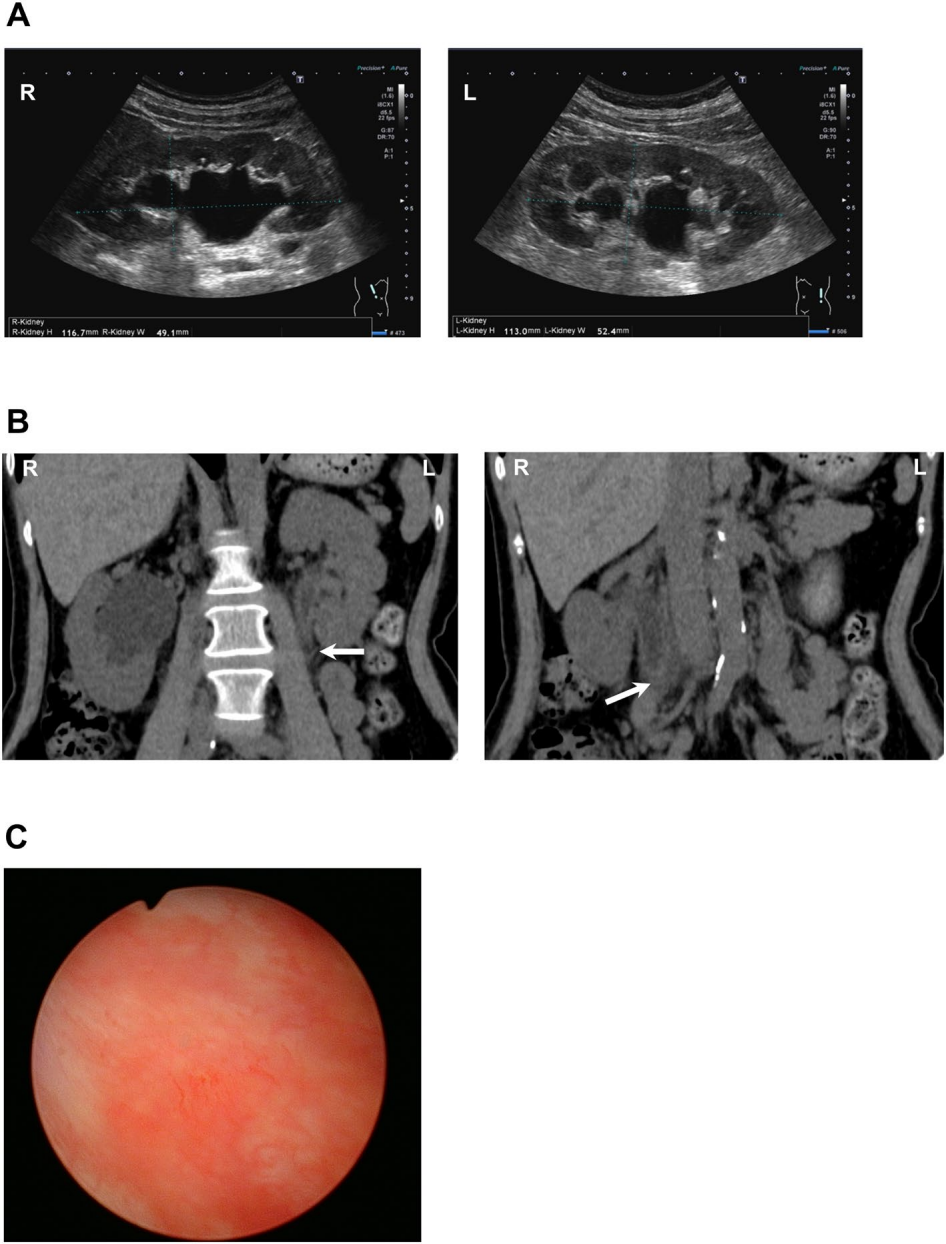
(A) Ultrasound imaging showing hydronephrosis of the (left image) right kidney and (right image) left kidney. (B) Computed tomography showing bilateral ureteral stenosis, as well as proximal ureteral strictures (arrows) with associated hydronephrosis in the bilateral upper urinary tract. (C) A Hunner lesion in the bladder.

**FIGURE 2:**
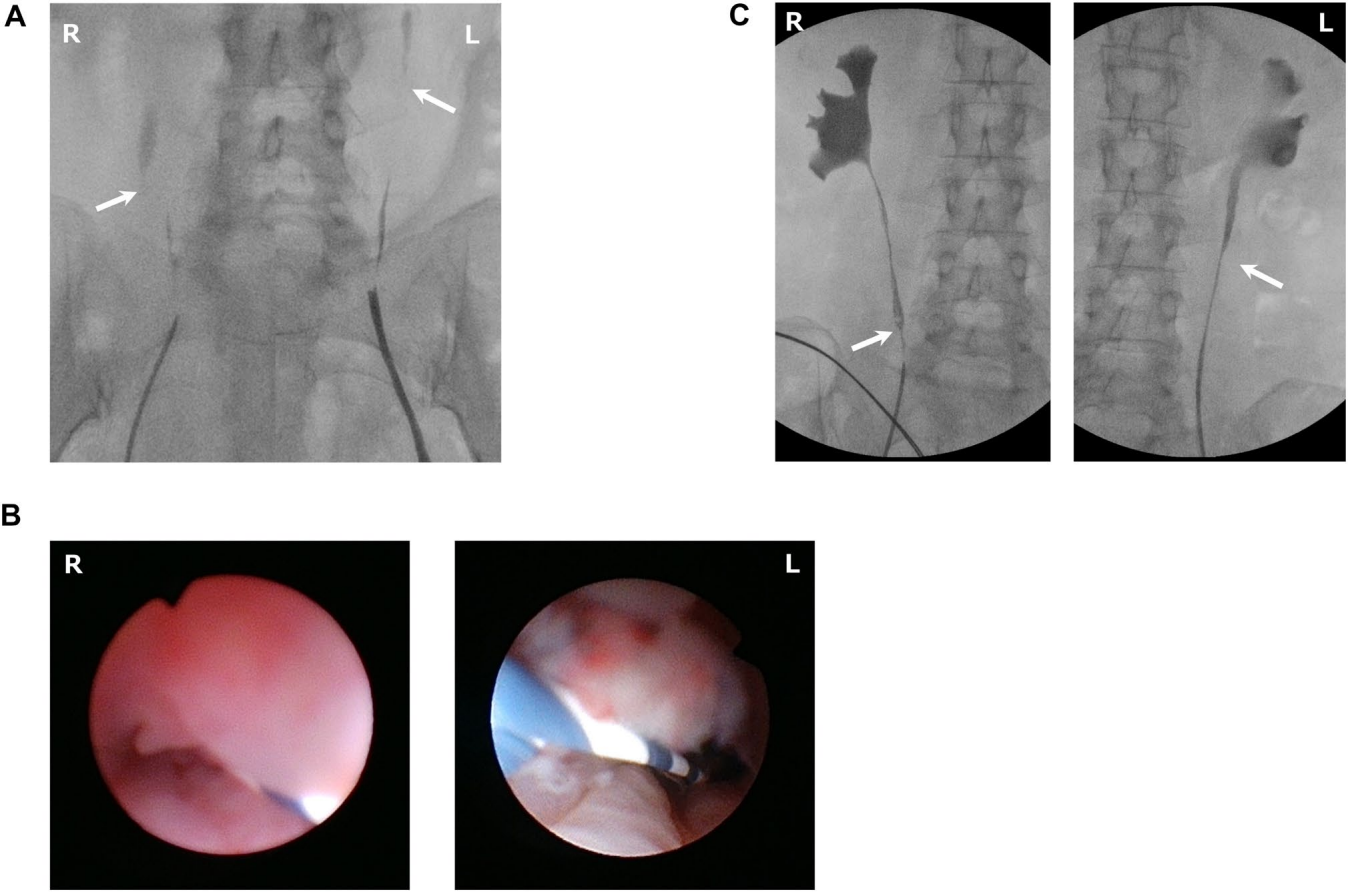
(A) Retrograde pyelography before corticosteroid therapy. Arrows indicate the bilateral proximal ureteral strictures. (B) Ureteroscopy before corticosteroid therapy, showing mucosal hyperplasia and luminal narrowing in the (left image) right ureter and (right image) left ureter. (C) Retrograde pyelography after 2 years of corticosteroid therapy. Arrows indicate the remaining bilateral ureteral stenosis and associated hydronephrosis.

The frequency volume chart showed that the maximum voided urine volume was 200 mL, consistent with a well‐preserved bladder capacity for a Japanese woman. Blood tests showed a serum creatinine concentration of 1.24 mg/dL and an estimated glomerular filtration rate (eGFR) of 36.3 mL/min/1.73 m^2^, suggestive of mildly impaired renal function. Concentrations of anti‐double stranded DNA and anti‐SS‐A antibodies were slightly elevated, but there was no evidence of comorbid systemic autoimmune disorders, such as systemic lupus erythematosus (SLE) and Sjogren's syndrome after a thorough examination by specialists in rheumatology and nephrology including histological analysis of renal biopsied tissue. Ultrasonography and CT imaging showed bilateral proximal ureteral narrowing and associated dilation of the upper ureters and pelvis, but no obvious mass forming lesions in the retroperitoneal space. Cystoscopy revealed the presence of a HL on the posterior bladder wall (Figure 1). She underwent endoscopic fulguration of the HL with simultaneous bladder hydrodistension, retrograde pyelography, ureteroscopy, and implantation of bilateral ureteral stents under general anesthesia. Bladder capacity at a pressure of 80 cm H_2_O was 350 mL. A retrograde pyelogram showed bilateral proximal ureteral narrowing and associated hydronephrosis, with subsequent ureteroscopy revealing hyperplasia of the ureteral mucosa at the narrowing sites (Figure 2). Neither a bladder deformity nor VUR was observed. After surgery, the patient's irritable IC/BPS symptoms were resolved, and bilateral hydronephrosis disappeared, leading to the normalization of her renal function. Then, her ureteral lesions were treated with oral prednisolone (7.5 mg/day) for 2 years, followed by retrograde pyelography and ureteroscopy to evaluate these lesions. Despite the relief of IC/HL symptoms after endoscopic surgery, her bilateral ureteral stenosis and associated hydronephrosis were not resolved after 2 years of corticosteroid therapy and the ureteral stents in both ureters were left in place (Figure 2).

## 3 Pathological Findings

**FIGURE 3:**
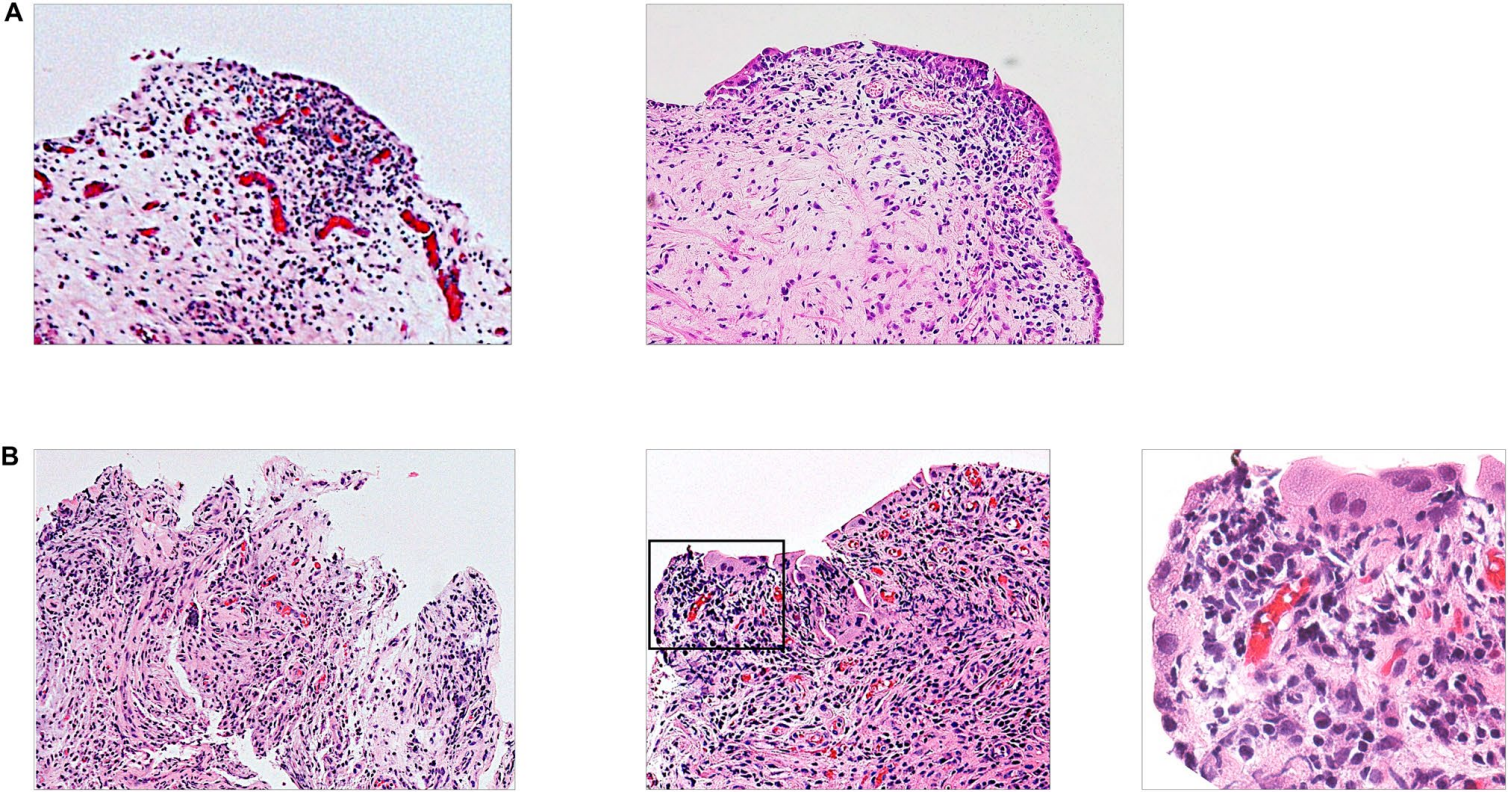
(A) Histology of bladder mucosal biopsies; (left image) the Hunner lesion, (right image) area outside the Hunner lesion. (B) Histology of ureteral lesions. Left image: Left ureter (original magnification, ×200), Middle image: Right ureter (original magnification, ×200), Right image: Enlarged image of the rectangular box in the middle image (original magnification, ×400).

Histological examination of the bladder biopsy specimens obtained from both the HL and an area outside the HL showed chronic inflammatory changes compatible with IC/HL, such as subepithelial infiltration of inflammatory cells, primarily lymphoplasmacytic cells, stromal edema and fibrosis, hyperemia, and epithelial denudation (Figure 3A) [6, 7]. Biopsy specimens obtained from the narrowing sites of bilateral ureters showed chronic inflammatory changes similar to those of the bladder specimens, including epithelial denudation and infiltration of lymphoplasmacytic cells into the subepithelial layer (Figure 3B). These findings suggest that the pathomechanisms responsible for the pathophysiology of the bladder and ureteral lesions were similar in this case.

## 4 Discussion

IC/HL has been reported to affect the entire bladder [8, 9], but the involvement of the upper urinary tract remains unclear. Results in the present patient, who developed bilateral ureteral IC/HL‐like inflammation and stenosis in addition to the bladder lesions despite the preservation of bladder capacity, suggest that IC/HL inflammation has involved the upper urinary tract.

IC/HL is an immune‐mediated inflammatory disease with a possible autoimmune nature [2, 3]. Epidemiologically, IC/HL is frequently accompanied by systemic autoimmune diseases such as SLE, Sjögren's syndrome, and Hashimoto's disease [4]. The elevated serum concentrations of autoantibodies, including anti‐SS‐A and anti‐double stranded DNA antibodies, in the present patient suggested the occurrence of other systemic autoimmune diseases. However, a thorough examination of this patient by specialists in rheumatology and nephrology resulted in no evidence of concurrent systemic autoimmune diseases, including SLE. Normal concentrations of the serum IgG4 antibodies and the absence of retroperitoneal fibrotic mass can negate the possible association of IgG4‐related disorders with ureteral stricture in this case. Taken together, these findings indicate that IC/HL alone was responsible for the elevated levels of serum autoantibodies and bilateral ureteral lesions in this case.

End‐stage IC/HL bladder can lead to bladder atrophy and deformity, with many of these patients experiencing bilateral/unilateral VUR and associated hydronephrosis [5]. To our knowledge, however, few studies have utilized ureteroscopy or histology to evaluate sporadic ureteral lesions potentially associated with IC/HL in bladders that were neither atrophied nor deformed. Findings in the present patient suggest that IC/HL could involve the upper urinary tract and impair kidney function at an earlier disease stage. Regular ultrasonographic or CT imaging follow‐up of upper urinary tracts in patients with IC/HL is warranted.

Low‐dose corticosteroid therapy was not effective in treating the ureteral lesions, although it sustained symptom relief after endoscopic elimination of the bladder lesions. Other immunosuppressants, including cyclosporine and tacrolimus, or more intensified prednisolone treatment, may be tested for the ureteral lesions of this case.

In conclusion, the present report describes a patient with IC/HL accompanied by bilateral ureteral stenosis and associated hydronephrosis. Histological assessment revealed chronic inflammatory changes in both the bladder and ureteral lesions compatible with those in typical IC/HL, suggesting that IC/HL inflammation had involved the upper ureters. Further investigations of additional patients are needed to clarify the pathomechanisms of unusual ureteral involvement in IC/HL.

## Ethics Statement

The present study was approved by the Institutional Review Board of the University of Tokyo (approval no. 3124). Written informed consent for participation was obtained from the patient. All procedures were performed according to the principles of the Declaration of Helsinki.

## Consent

All authors have read the entire manuscript and agreed to publication.

## Conflicts of Interest

Tomonori Minagawa and Yusuke Sato are Editorial Board members of the International Journal of Urology and co‐authors of this article. To minimize bias, they were excluded from all editorial decision‐making related to the acceptance of this article for publication. Other authors declare no conflicts of interest with respect to the authorship and publication of this article.

## Data Availability

All data presented in this study are available within the article or from the corresponding author upon reasonable request.
